# WDR4-mediated m7G modification of ZBP1 mRNA drives peri-implantitis by promoting M1 macrophage polarization

**DOI:** 10.1186/s41065-026-00723-5

**Published:** 2026-08-14

**Authors:** Xuexia Chen, Yanping Jiang, Haibin Lu

**Affiliations:** Stomatological Hospital, School of Stomatology, Southern Medical University, S366 Jiangnan Boulevard, Haizhu District, Guangzhou City, Guangdong Province China

**Keywords:** WDR4, m7G modification, M1 macrophage, Peri-implantitis

## Abstract

**Objective:**

Macrophage polarization exerts a vital role in the progression of peri-implantitis. RNA methylation serves as an important regulator of macrophage polarization. However, reports on the role of N7-methylguanosine (m7G) regulators in peri-implantitis are scarce.

**Methods:**

The function of m7G regulators in peri-implantitis and macrophage polarization was investigated via in vivo and in vitro experiments. RNA sequencing (RNA-seq) and m7G methylated RNA immunoprecipitation sequencing (m7G MeRIP-seq), and molecular biology assays were performed to identify and validate downstream m7G-modified target genes.

**Results:**

WDR4 was identified as the sole significantly upregulated m7G-related gene in peri-implantitis tissues. Overexpression of WDR4 promoted macrophage M1 polarization, which further inhibited osteogenic differentiation of bone marrow mesenchymal stem cells (BMSCs). RNA-seq revealed 3001 differentially expressed genes (DEGs) in WDR4-overexpressing macrophages, including 1503 upregulated genes and 1498 downregulated genes. m7G MeRIP-seq identified 554 m7G differential peaks between the control and WDR4 overexpression group. Integrative analysis of RNA-seq and m7G-MeRIP-seq identified 33 genes with both increased expression and enhanced m7G methylation, among which ZBP1 was selected as a key downstream target. WDR4 interacted with ZBP1 mRNA, increased its m7G modification, and enhanced its mRNA stability. Silencing ZBP1 significantly attenuated WDR4-induced M1 macrophage polarization and restored the impaired osteogenic differentiation of BMSCs.

**Conclusions:**

WDR4 promotes M1 polarization of macrophages by enhancing the m7G modification of ZBP1, thereby inhibiting BMSC osteogenic differentiation and promoting peri-implantitis progression. Targeting the WDR4/m7G/ZBP1 axis may represent a potential therapeutic strategy for peri-implantitis.

**Supplementary Information:**

The online version contains supplementary material available at https://doi.org/10.1186/s41065-026-00723-5.

## Introduction

Peri-implantitis is an inflammatory disease characterized by progressive bone loss and inflammation of the connective tissue around the implant [1]. Peri-implantitis is widespread, with a weighted average prevalence of 43% in Europe and 22% in South and North America [2]. While bacterial biofilms are the primary etiological factor, there are many factors that influence the occurrence and progression of the disease. Factors affecting the development of peri-implantitis include a history of periodontitis, inadequate plaque control, failure to undergo regular periodontal maintenance, poor spatial orientation of implant fixtures, and the lack of keratinized mucosa [3]. Peri-implantitis significantly threatens osseointegration between the alveolar bone and the implant, which is crucial for the long-term stability of the implant [4]. Clinically, guided bone regeneration techniques are recommended to fill bone defects caused by peri-implantitis and to restore contact between the bone and the implant. The success and reliability of this treatment approach are limited, partly because the behavior of local osteoblasts is disrupted in the immune-inflammatory microenvironment [5]. Therefore, exploring the mechanisms of inflammation is crucial for the treatment of peri-implantitis.

Macrophages are important inflammatory cells that play a role in anti-infection, regulation of immune responses, and maintenance of internal environment stability. They can be polarized to distinct phenotypes, M1 or M2, in reaction to diverse biophysical and biochemical signals. M1 macrophages function in pro-inflammatory responses, while M2 macrophages are involved in inflammation resolution and tissue repair [6, 7]. Pathological studies have found that peri-implantitis lesions are larger, with a greater number and ratio of macrophages and vascular infiltrates around the implants [8]. Macrophages have a dual role in peri-implantitis, contingent upon their phenotype. M2 macrophages can contribute to osseointegration and effective wound healing, while M1 macrophages can intensify the inflammatory process and facilitate bone resorption, which results in implant failure. Compared to samples from periodontal diseases, an increase in the population of M1 macrophages was observed on peri-implantitis samples [9]. In recent years, strategies to prevent and even treat peri-implantitis by regulating macrophage polarization have received increasing attention. Zhang et al. were the first to prepare L-MOF-agomir implants and found that these implants effectively promoted the polarization of rat macrophages from M1 to M2, promoted bone regeneration, and reduced inflammation and bone resorption around the implants caused by ligation [10]. This suggests that the polarization of macrophages is a key factor in the progression of peri-implantitis.

The N7-methylguanosine (m7G) modification is one of the most familiar forms of RNA modification in post-transcriptional regulation, distributed at the 5′cap and internally in tRNA, rRNA, and mRNA. It plays a momentous part in various biological processes like transcription elongation, mRNA splicing, and mRNA translation [11]. METTL1, together with its cofactor WDR4, catalyzes the m7G modification of various types of RNAs [12]. m7G modification has been reported to participate in human disease development, such as neurodegenerative disorder, cancer, and cardiovascular disease. METTL1 facilitated the development of esophageal squamous cell carcinoma through the RPTOR/ULK1/autophagy pathway [13]. METTL1 enhanced SRSF9 levels via m7G modifications of SRSF9 mRNA, which accelerated the alternative splicing and stabilization of NFATc4, ultimately leading to cardiac hypertrophy [14]. However, the role of m7G modification in peri-implantitis remains unclear.

This study intends to illustrate the role and related mechanisms of WDR4-mediated m7G modification in peri-implantitis through macrophage polarization using cell, animal, and molecular experiments. We uncovered that WDR4 drives M1 macrophage polarization through m7G modification, which in turn inhibits BMSC osteogenesis and promotes the advancement of peri-implantitis. The research offers novel insights for the treatment of peri-implantitis.

## Materials and methods

### Peri-implantitis mouse model

Twelve 4-week-old male C57BL/6J mice were bought from Beijing SPF Biotechnology Co., Ltd (China). The study protocols were endorsed by the Institutional Committee for Animal Use and Care at the university. Throughout the experiment, the mice had unrestricted access to a soft diet. Following a 1-week adaptation period, the mice were randomly allocated to two groups: a control group, a peri-implantitis model group, with 6 mice in each group. For the model construction [15], intraperitoneal injection of avertin (200 µl/10 g) was used to anesthetize mice, and all surgical procedures were performed using a surgical magnifying glass under standard sterile conditions. The left maxillary first molar and the corresponding first molar on the left mandible were extracted using micro-tissue forceps. Subsequently, a custom driver was used to immediately implant a pure titanium spiral-shaped implant (material: grade 4 titanium; length 1.7 mm, main diameter 0.7 mm, tip 0.4 mm, external hexagon side 0.9 mm) into the distal palatal root socket left by the extraction of the left maxillary molar. To prevent infection in the first three days postoperatively, 10 mg of penicillin was injected into the mice’s abdominal cavity daily. After surgery, the mice were fed a soft diet and sterile water for 4 weeks to allow for osseointegration of the implant. Following the healing of the implants, the 5 − 0 silk thread soaked in heat-inactivated *Porphyromonas gingivalis* was tightly wrapped around the subgingival cervical part of the implant in the model group for 2 turns to induce peri-implantitis. No ligatures were applied to the implants in the control group. After 2 weeks of feeding, maxillary implants were photographed. Then the mice gingival tissues and maxillae were obtained after the mice sacrificed by CO2 inhalation. Bone resorption was measured by microscopic imaging analysis and micro-computed tomography (µCT) scanning analysis.

### Hematoxylin and eosin (HE) staining

Tissue sections were baked at 65 °C for 30 min, deparaffinized in xylene, and rehydrated through graded ethanol. After washing, sections were stained with hematoxylin for 5 min, differentiated in 1% acid alcohol, and blued in saturated lithium carbonate. The sections were dehydrated in 95% ethanol, stained with eosin, and sequentially dehydrated in 95% and 100% ethanol. Finally, sections were cleared in phenol-xylene and xylene, mounted with neutral balsam, and examined microscopically.

### Tartrate-resistant acid phosphatase (TRAP) staining

Following deparaffinization and rehydration, tissue sections were circled with a hydrophobic pen and pre-incubated with distilled water at 37 °C for 2 h in a humidified chamber. The sections were incubated with TRAP incubation solution at 37 °C for 20 min. After washing, the sections were counterstained with hematoxylin for 15 s. Finally, the sections were dehydrated, cleared, mounted with neutral balsam, and examined microscopically.

#### m7G dot blot

The extracted total RNA was denatured at 95 °C for 3 min, and transferred to a nitrocellulose membrane (F619512-0001, Sangon Biotech, China). After washing the membrane with Wash Buffer to remove unbound RNA, the membrane underwent incubation in Blocking Buffer for 1 h at ambient temperature. Afterwards, the membranes were probed with an anti-m7G antibody (RN017M, MBL, Japan) at room temperature for 2 h, followed by incubation with a secondary antibody (A0208,　Beyotime, China)　for one hour. Finally, chemiluminescence was carried out using the ECL chemiluminescence kit (KF003, Affinity, USA), and the chemiluminescence effect was recorded using the chemiluminescence imaging system.

### PCR array of RNA modification-related genes

RNA was isolated from gingival tissues of control and model groups using the RNeasy Mini Kit (Qiagen, 74104). Subsequently, the conversion of total RNA into cDNA was carried out using the RT2 First Strand Kit (Qiagen, 330401). The epitranscriptomics PCR Array and quantitative real-time PCR (qRT-PCR) were utilized to assess mRNA levels as per the supplier’s guidelines. qRT-PCR was implemented on the ABI 7500 Real-Time PCR System (Thermo Fisher, United States) by utilizing the RT2 SYBR Green Master mix (Qiagen, 330401) in accordance with the manufacturer’s directions. The 2-ΔΔCt method was employed to examine the expression of RNA modification-related genes.

### Cell culture

THP-1 cells and bone marrow mesenchyml stem cells (BMSCs) were procured from iCell Bioscience Technology Co., Ltd (Shanghai, China). THP-1 cells were maintained in RPMI 1640 medium (10-040-CVR, CORNING), which contained 1% penicillin/streptomycin and 10% fetal bovine serum. BMSCs were cultured in BMSCs-specific culture medium (PriMed-iCell-012-SF, iCell). Cells were cultivated at 37 °C with 5% CO2 in a humidified incubator.

THP-1 cells were differentiated into macrophages using phorbol 12-myristate 13-acetate (PMA) at a concentration of 100 ng/ml. To induce M1 polarization, macrophages were cultured for 48 h with 100 ng/ml PMA, 100 ng/ml lipopolysaccharide (LPS), and 20 ng/ml interferon-γ (IFN-γ).

### Construction of the co-culture system of macrophages and BMSCs

The co-culture chambers were embedded in 24-well plates to construct a co-culture system of BMSCs (lower chamber) and M1 macrophages (upper chamber) at a ratio of 1:1. Then, 1 ml of RPMI 1640 complete medium was added to the upper chamber, and 300 µl of mesenchymal stem cell osteogenic induction differentiation medium (iCell-MSCYD-002, iCell) was added to the lower chamber. The 24-well plate was replaced every 3 days to provide freshly induced M1 macrophages. After co-culture for 7 days, BMSCs were collected for RNA extraction.

### Cell transfection

WDR4 over-expressing vector, empty vector, siZBP1, siNC, WDR4 wild-type and mutant constructs were provided by GenePharma (Shanghai, China). Plasmids and siRNAs were introduced into macrophages utilizing Lipofectamine 2000 Transfection Reagent (1166019, Invitrogen), followed by various assays.

### Immunofluorescence

For mouse gingival tissue immunofluorescence, paraffin-embedded gingival tissue sections were deparaffinized, rehydrated, and subjected to antigen retrieval in citrate buffer. After permeabilization with 0.3% Triton X-100 and blocking with 5% bovine serum albumin (BSA), the sections were incubated with primary antibodies against CD86 (105001, BioLegend), and against WDR4 (11427-1-AP, Proteintech) overnight at 4 °C, followed by Alexa Fluor-conjugated secondary antibodies (A11006 and A11012, Invitrogen) for 1 h at room temperature. Nuclei were counterstained with DAPI.

Macrophages were immobilized using 4% paraformaldehyde, rinsed three times with PBS, followed by a blocking step with with 5% BSA for half an hour. Next, they were incubated with primary antibodies against CD86 (1:500, PA5-88284, Invitrogen) at 4 °C for an overnight period. Afterward, the cells were incubated with a Cy3-labeled secondary antibody (1:200, A0516, Beyotime) at room temperature for one hour, followed by a 10-minute staining with DAPI. The fluorescence were observed with fluorescence microscopy (CYX53, OLYPUS).

### Quantitative real-time PCR (qRT-PCR)

RNA isolation was carried out with Trizol reagent as per the supplier’s manual. Reverse transcription was executed with 1 mg of RNA from each sample to generate first-strand cDNA using the PrimeScript RT Master Mix (Thermo). qRT-PCR was conducted on the ABI Q6 system (Applied Biosystems Inc.USA) with SYBR Green PCR Master Mix (Roche). The relative mRNA expression of WDR4, CD86, MCP-1, iNOS, OPN, RUNX2, BMP2, ALP, and ZBP1 was assessed using the 2^-ΔΔCt method, normalized to GAPDH. The primer sequences are provided in Table S1.

### Western blot

Cell lysates were prepared with RIPA buffer, and protein concentrations were measured utilizing a BCA protein assay kit (Beyotime). Protein were fractionated by 10% SDS-PAGE, after which the proteins were transferred to PVDF membranes. The membranes were then soaked in a 5% non-fat milk at room temperature for one hour. Whereafter, the membranes were probed with primary antibodies against WDR4 (1:500, Sc-100894, Santa), and GAPDH (1:5000, 60004-1-lg, Proteintech) overnight at 4 °C. The membranes were then incubated with HRP-conjugated secondary antibodies Goat Anti-Mouse IgG H&L(1:5000, RGAM001, Proteintech) or Goat Anti-Rabbit IgG H&L(1:5000, RGAR001, Proteintech) for one hour. Afterward, protein bands underwent color development with an ECL reagent. The intensity of the bands was analyzed with ImageJ software to determine the grayscale values.

### Alkaline phosphatase (ALP) staining

ALP activity was detected utilizing the BCIP/NBT ALP Color Development Kit (Beyotime, C3206) based on the supplier’s protocol. In detail, cells were fixed with 4% paraformaldehyde for 20 min. The BCIP/NBT working solution was prepared freshly according to the manufacturer’s instructions. After removing the PBS, the working solution was added to fully cover the samples, followed by incubation in the dark at room temperature for 2 h. Microscopic visualization of the results was achieved using a microscope.

### RNA sequencing (RNA-seq)

To identify the differentially expressed genes (DEGs) in WDR4 overexpressed and control macrophages, we performed RNA-seq. The construction of the cDNA Library of the samples were conducted by Shanghai MivectorBio Company. The cDNA libraries underwent sequencing in paired-end mode with 150 bp reads on the Illumina HiSeq 2500 platform (USA). In the data analysis stage, the FastQC software was applied to perform an overall quality assessment of the sequencing data. DEGs were screened with the aid of the DESeq2 package, and the absolute value of log2FC > 1 and FDR < 0.05 were set as the screening threshold. Finally, functional analysis of these DEGs were conducted using Gene Ontology (GO) and KEGG pathway analysis. The Fisher’s exact test was employed to calculate the significance level (P-value) for each GO term or pathway. Subsequently, multiple hypothesis testing correction was employed to obtain the FDR, allowing for the identification of significantly enriched GO terms or pathways associated with DEGs.

### m7G methylated RNA immunoprecipitation sequencing (m7G MeRIP-seq)

The MeRIP-seq experiments and subsequent data analysis were facilitated by MivectorBio (Shanghai, China). The total RNA from macrophages (18µL) was fragmented using 10µL of RNA Fragmentation Buffer and then processed in a PCR machine at 70 °C for 10 min. Post-reaction, the PCR tubes were chilled on ice. The total volume of the samples was adjusted to 180 µL by adding nuclease-free water. Thereafter, 18 µL of 3 M sodium acetate, 2 µL of glycogen, and 600 µL of absolute ethanol were added. The samples were centrifuged at 18,000 rpm and 4 ° C for 30 min, followed by removal of the supernatant. The anti-m7G antibody (4µL) was pre-incubated with 50µL of A/G magnetic beads in 100µL IP buffer at room temperature for 30 min. The 200µL of MeRIP reaction mixture, 20µL of 10% SDS, 5µL of ProteinaseK (10 mg/ml) were then appended to the prepared m7G magnetic beads and incubated at 55 °C for 60 min. Afterward, 225µL of acidic phenol: chloroform: isopropanol was appended to the sample, and the mixture was centrifuged at 10,000 g at room temperature for 5 min. The upper aqueous phase was transferred to a new tube, added 500µL of isopropyl alcohol and 2µL of glycogen, and incubated at room temperature for 3 min. After centrifuging at 4 °C and 20,000 g for 30 min, 10 µL of DEPC water was appended to dissolve the precipitate. Finally, MeRIP-seq was performed by MivectorBio (Shanghai, China). The DREME software was used for motif analysis of peaks. Differentially RNA methylated sites were screened based on Fold change more than 10 and P-value less than 1e-05. Functional analysis of m7G differential genes were conducted using GO and KEGG pathway analysis.

### RNA immunoprecipitation (RIP) assay

Cells were lysed in RIP Lysis Buffer and stored at -80 °C. Meanwhile, magnetic beads were coupled with 5 µg of anti-WDR4 antibody (sc-100894, Santa) or 1 µg of IgG control for 30 min at room temperature. After clearing the lysates by centrifugation, 100 µL of supernatant was incubated with the bead-antibody complexes in 1 mL of RIP Immunoprecipitation Buffer overnight at 4 °C. The complexes were washed 6 times with RIP Wash Buffer, digested with Proteinase K at 55 °C for 30 min, and the co-precipitated RNA was extracted using TRIzol. The isolated RNA was finally analyzed by qRT–PCR.

### m7G MeRIP-PCR

Total RNA was extracted using TRIzol reagent according to the manufacturer’s instructions. RNA was fragmented into approximately 100–200 nt fragments using RNA fragmentation buffer. Subsequently, fragmented RNA was incubated with anti-m7G antibody and immunoprecipitated using Protein A/G magnetic beads. After washing, the immunoprecipitated RNA was eluted and purified for reverse transcription. Enrichment of m7G-modified transcripts was determined by qRT-PCR.

### mRNA stability assay

To assesss ZBP1 mRNA stability in macrophages, we treated the cells with 5 µg/mL of actinomycin D for 0, 2, 4, 6, and 8 h. Subsequently, total RNA was isolated and subjected to qRT-PCR to evaluate the ZBP1 mRNA stability.

### Statistical analysis

Results are presented as mean ± standard deviation. Statistical analysis was conducted using GraphPad Prism 9.0 software. Differences between two groups were assessed using the Student’s t-test, while one-way ANOVA followed by Tukey’s post-hoc test was applied for multiple group comparisons. The Shapiro-Wilk test and Levene’s test were used to test for normality and homogeneity of variance. A threshold of *p* < 0.05 indicated statistical significance.

## Results

### WDR4 is upregulated in peri-implantitis tissues

**Fig. 1:**
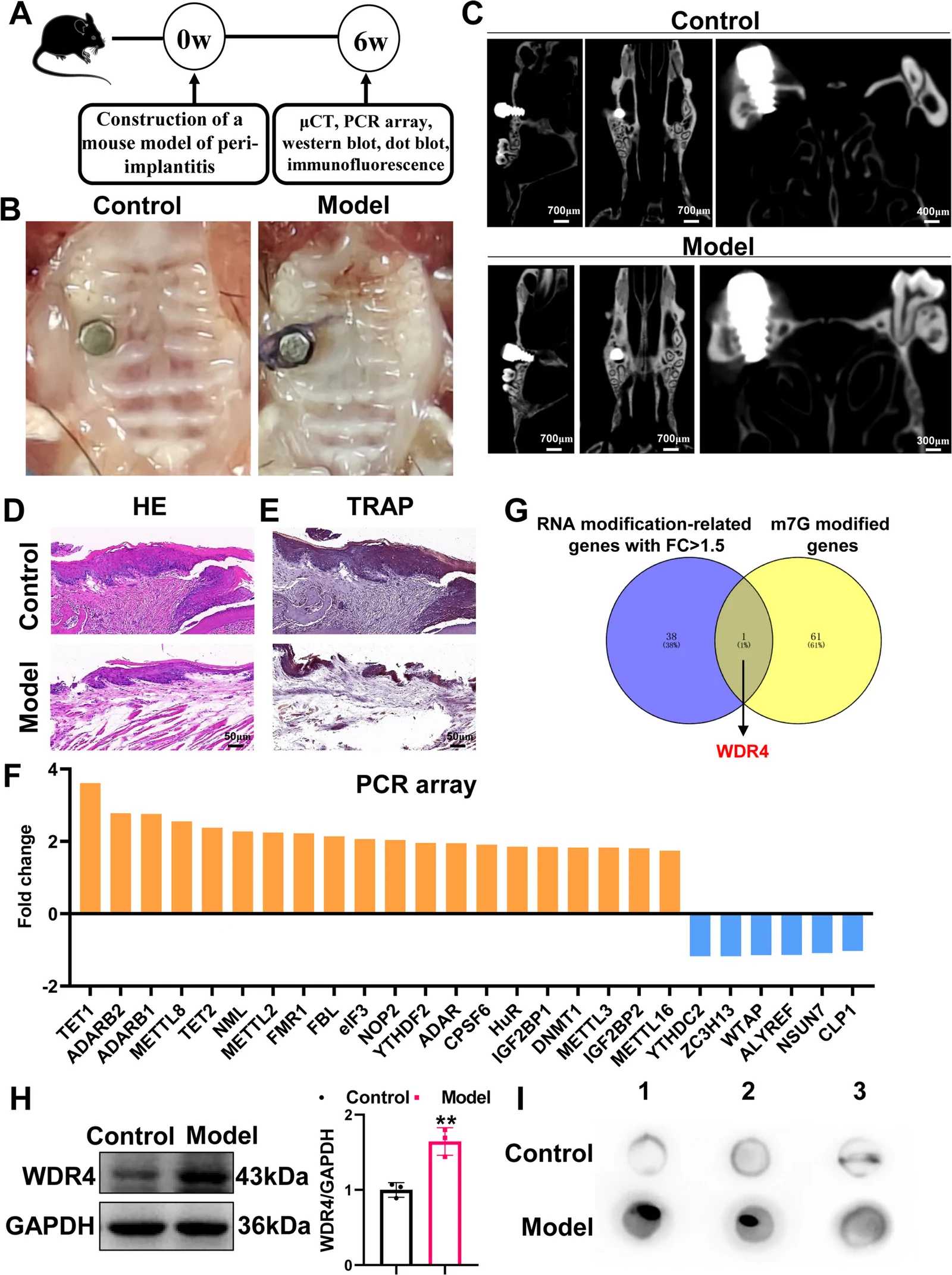
WDR4 is upregulated in peri-implantitis tissues. **A** Flow diagrams of animal experiments. **B** Observation of the gingiva around the implants of mice in the control and model groups. **C** Micro-CT results of mouse implants. **D** Representative HE staining of peri-implant tissues. **E** Representative TRAP staining showing osteoclast accumulation around peri-implant bone. **F** Epigenetic modification PCR array results. **G** Venn analysis between RNA modification-related genes with FC > 1.5 and m7G-releated genes. **H** Western blot analysis of WDR4 expression in gingival tissues of the control and model groups. **I** m7G dot blot. Data are presented as mean ± SD (*n* = 3). **P < 0.01

To investigate whether m7G modification regulates peri-implantitis, we first established a mouse model of peri-implantitis (Fig. 1A). In the control group, no swelling or exudation was observed around the implant, whereas the model group exhibited significant swelling around the implant (Fig. 1B). Micro-CT results showed that the model group had obvious alveolar bone resorption, while the control group showed almost no resorption (Fig. 1C). HE staining showed pronounced inflammatory cell infiltration and destruction of peri-implant tissues in the model group (Fig. 1D). Consistently, TRAP staining demonstrated a marked increase in osteoclast accumulation surrounding peri-implant bone in peri-implantitis mice (Fig. 1E), indicating enhanced osteoclastic bone resorption. Next, we collected gingival tissues from the model and control group mice and performed epigenetic modification PCR array analysis. The expressions of differentially RNA modification-related genes were shown in Fig. 1F. To identify m7G modification-related genes that regulate peri-implantitis, we performed a Venn analysis of DEGs with fold change > 1.5 and a gene set associated with m7G modification. The results revealed only one intersecting gene, WDR4 (Fig. 1G), which was prominently upregulated in the peri-implantitis group relative to the control group. This result was further validated by western blot (Fig. 1H). m7G dot blot results uncovered that the model group exhibited a higher level of m7G modification relative to the control group (Fig. 1I). Overall, the expression of WDR4 was elevated in peri-implantitis tissues.

#### WDR4 inhibits the osteogenic differentiation of BMSCs via promoting the M1 polarization of macrophage

**Fig. 2:**
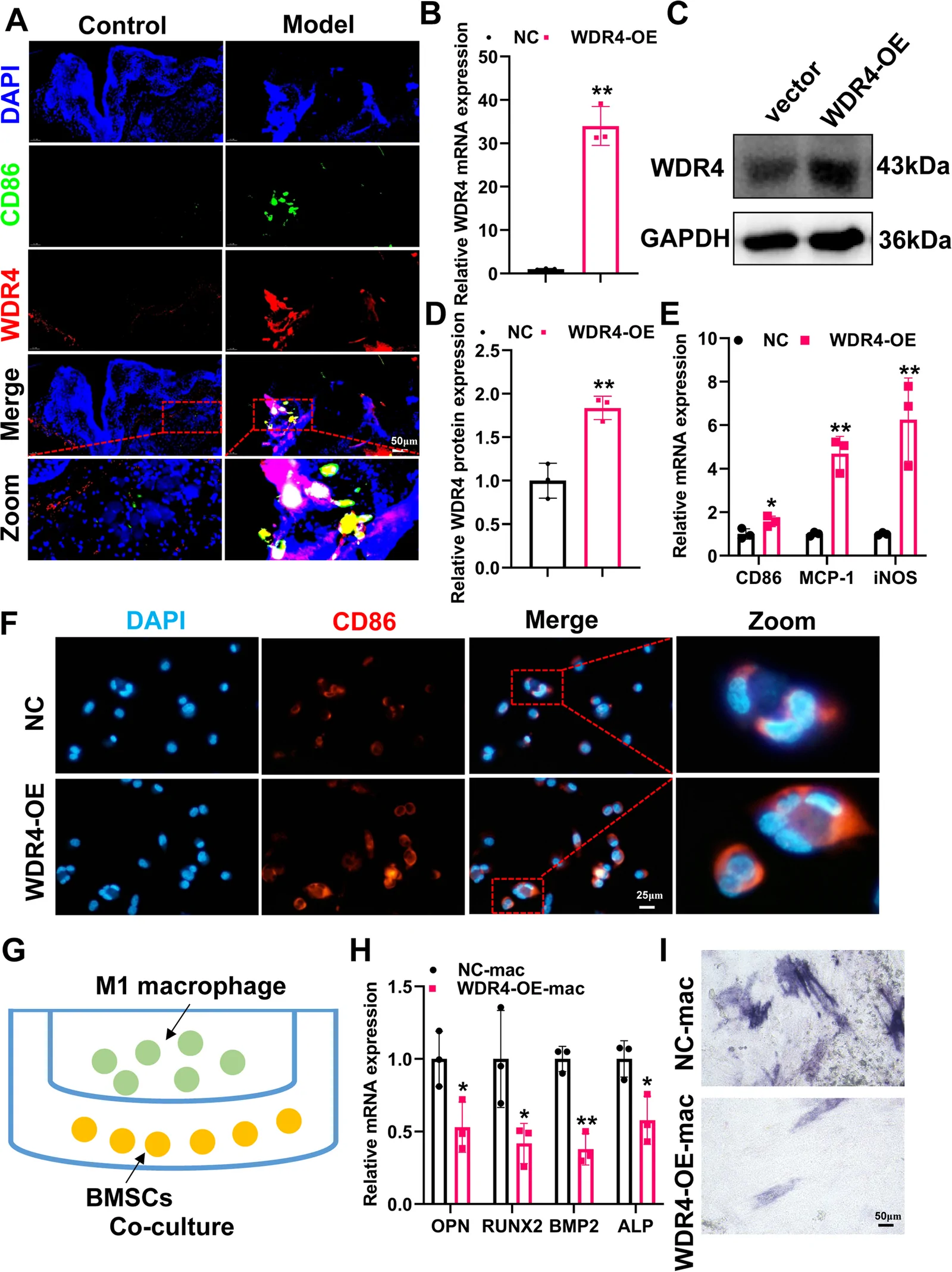
WDR4 suppresses the osteogenic differentiation of BMSCs by facilitating the polarization of macrophages towards the M1 phenotype. **A** Immunofluorescence detection of the co-localization of CD86 and WDR4 in gingival tissue. Scale bar=50 μm. **B**-**D** qRT-PCR **B** and western blot **C**, **D** were applied to assess the efficacy of WDR4 overexpression. **E** The expression of M1 markers (CD86, MCP-1, and iNOS) in WDR4-overexpressed and control macrophages was examined by qRT-PCR. **F** The expression of CD86 was assessed by immunofluorescence. Scale bar=25 μm. **G** Model diagram of co-culture of M1 macrophages and BMSCs. **H** qRT-PCR was utilized to measure the expression of osteogenic differentiation markers. **I** ALP staining. Data are presented as mean ± SD (*n* = 3). *P < 0.05, **P < 0.01

To investigate the function of WDR4 on macrophage polarization, we first examined the co-expression of CD86 and WDR4. The immunofluorescence results disclosed that the expressions of CD86 and WDR4 in the gingival tissues were increased and were co-localized in the model group (Fig. 2A). Subsequently, the WDR4 overexpression vector was transfected into macrophage and the overexpression efficiency of WDR4 was confirmed by qRT-PCR and western blot (Fig. 2B-D). qRT-PCR results showed that overexpression of WDR4 notably increased the expression of M1 markers (CD86, MCP-1, and iNOS) in macrophages (Fig. 2E). Immunofluorescence results indicated that overexpression of WDR4 promoted the expression of CD86 in macrophages (Fig. 2F). The above findings suggest that WDR4 facilitates M1 polarization of macrophages. To further investigate the role of WDR4-mediated M1 macrophage polarization in the osteogenic differentiation of BMSCs, we co-cultured BMSCs with macrophages overexpressing WDR4 (Fig. 2G). qRT-PCR results demonstrated that osteogenic differentiation markers OPN, RUNX2, BMP2, and ALP were observably downregulated in the WDR4 overexpression group relative to the control group (Fig. 2H). ALP staining showed that co-culture with WDR4-overexpressing macrophages decreased ALP activity of BMSCs (Fig. 2I). Overall, these findings indicated that WDR4 represses the osteogenic differentiation of BMSCs through M1 macrophage polarization.

#### Identification of target genes of WDR4 in macrophages

**Fig. 3:**
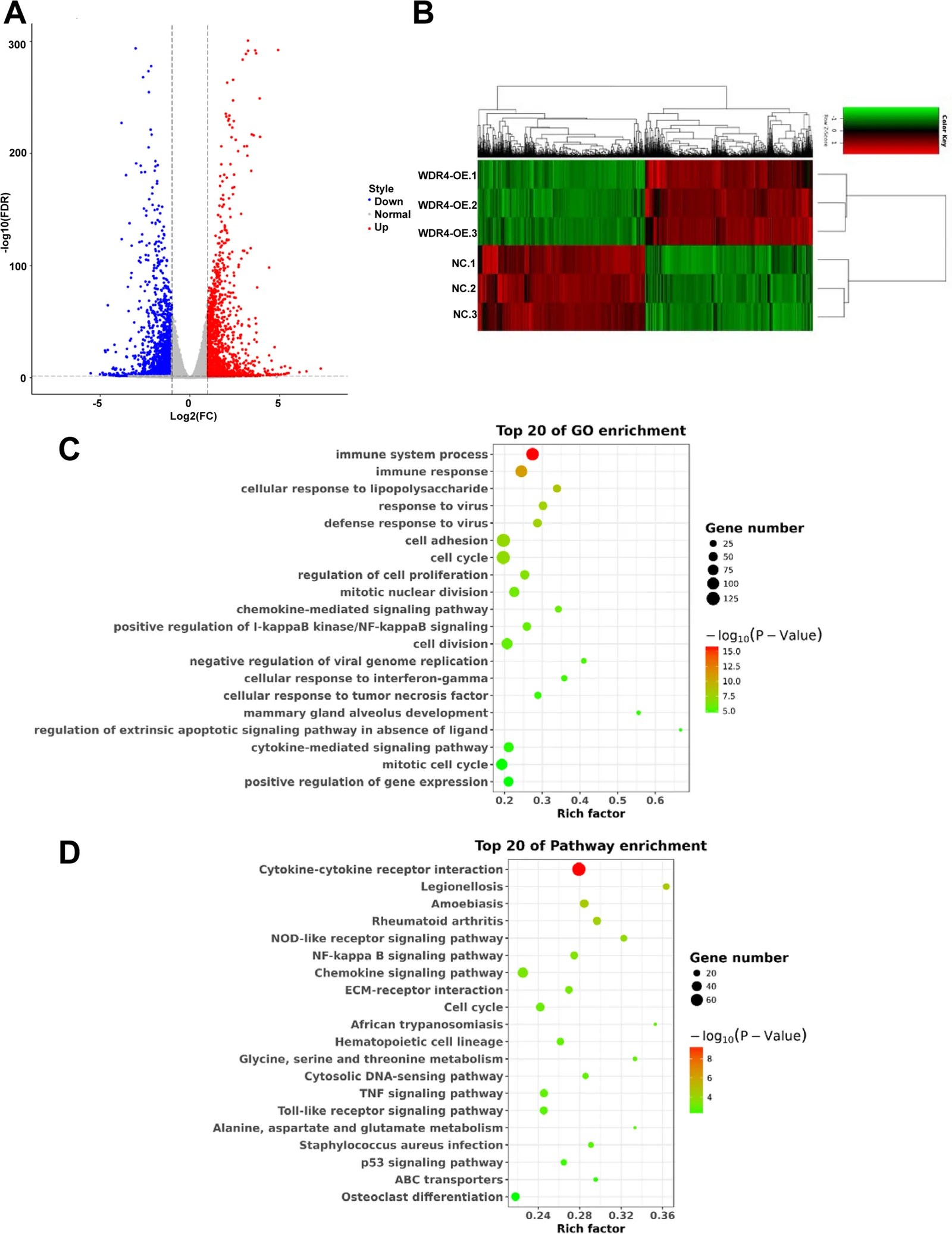
Differential expression and functional analysis of WDR4 target genes. **A**, **B** Volcano plot (**A**) and heatmap (**B**) of differentially expressed genes in macrophages after WDR4 was overexpressed. **C**, **D** GO (**C**) and KEGG (**D**) analysis of WDR4 target genes

To elucidate the molecular mechanism by which WDR4 modulate macrophage polarization, we conducted RNA-seq on macrophages with WDR4 overexpression and control macrophages. Volcano plot and heatmap showed that in comparison with the control group, there were 3001 DEGs in the WDR4 overexpression group, among which 1503 genes were upregulated and 1498 genes were downregulated (Fig. 3A and B). GO analysis revealed that DEGs were primarily engaged in immune system processes, immune responses, and cell adhesion (Fig. 3C). KEGG pathway analysis manifested that DEGs principally participated in the NOD-like receptor, NF-κB, and TNF signaling pathways (Fig. 3D). Overall, WDR4 targeted genes were associated with immune responses.

#### Analysis of the m7G modification gene profile regulated by WDR4

**Fig. 4:**
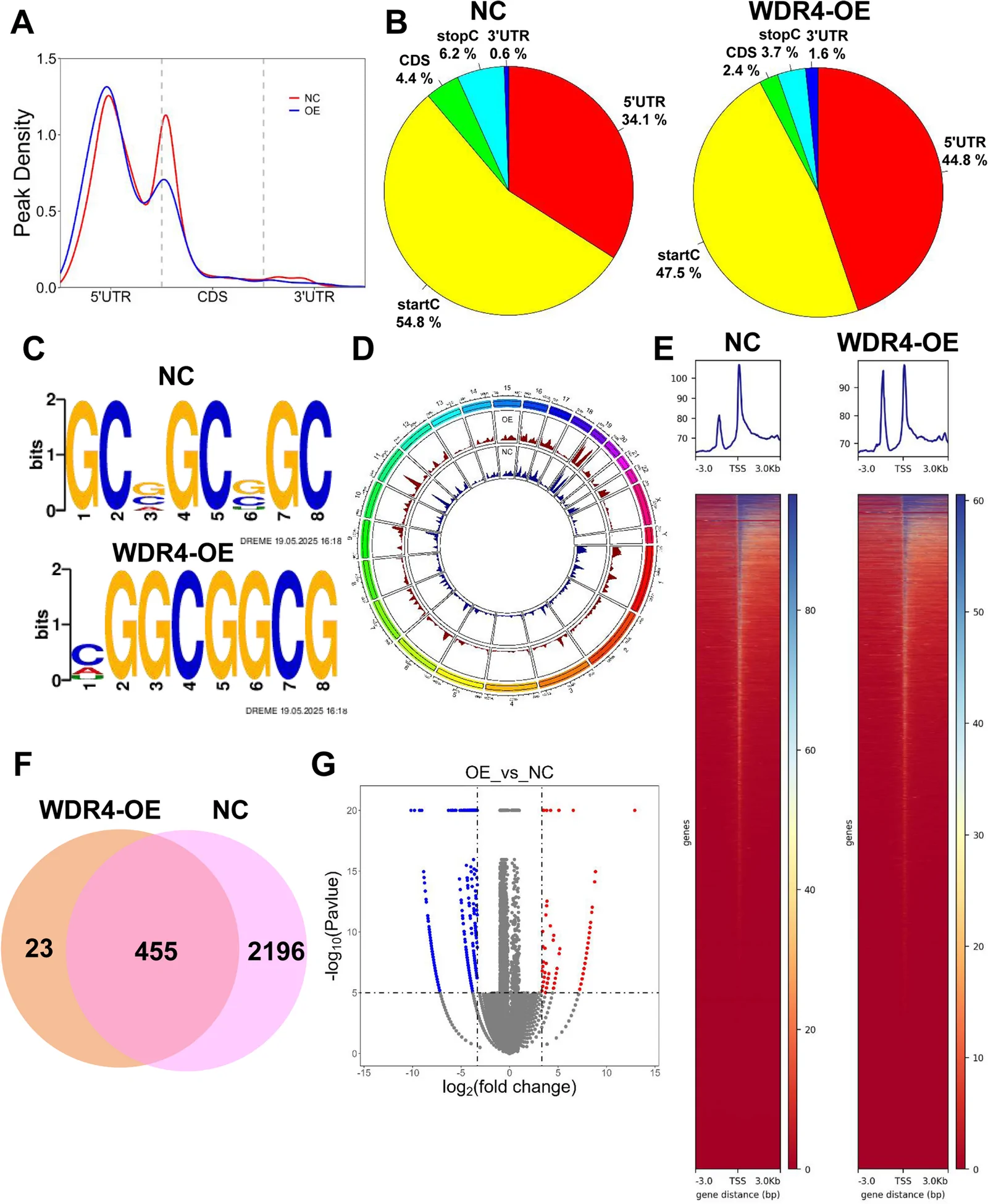
A comparison of m7G modification profiles between the control and WDR4 overexpression groups. **A** The distribution of m7G peak density. **B** The distinct patterns of occupancy at coding sequences, stop codons, 5’ and 3’ untranslated regions, and start codons. **C** Motif sequences of the control and WDR4 overexpression groups. **D** The Circos plot revealed the distribution of m7G modification peaks in the control and WDR4 overexpression groups. **E** Heatmap illustrated the intensity of the m7G modification signal in proximity to the TSS within the transcriptome. **F** Venn plot depicts common genes with m7G modification between the control and WDR4 overexpression groups. **G** Volcano plot of differential m7G peaks in macrophages after WDR4 overexpression

**Fig. 5:**
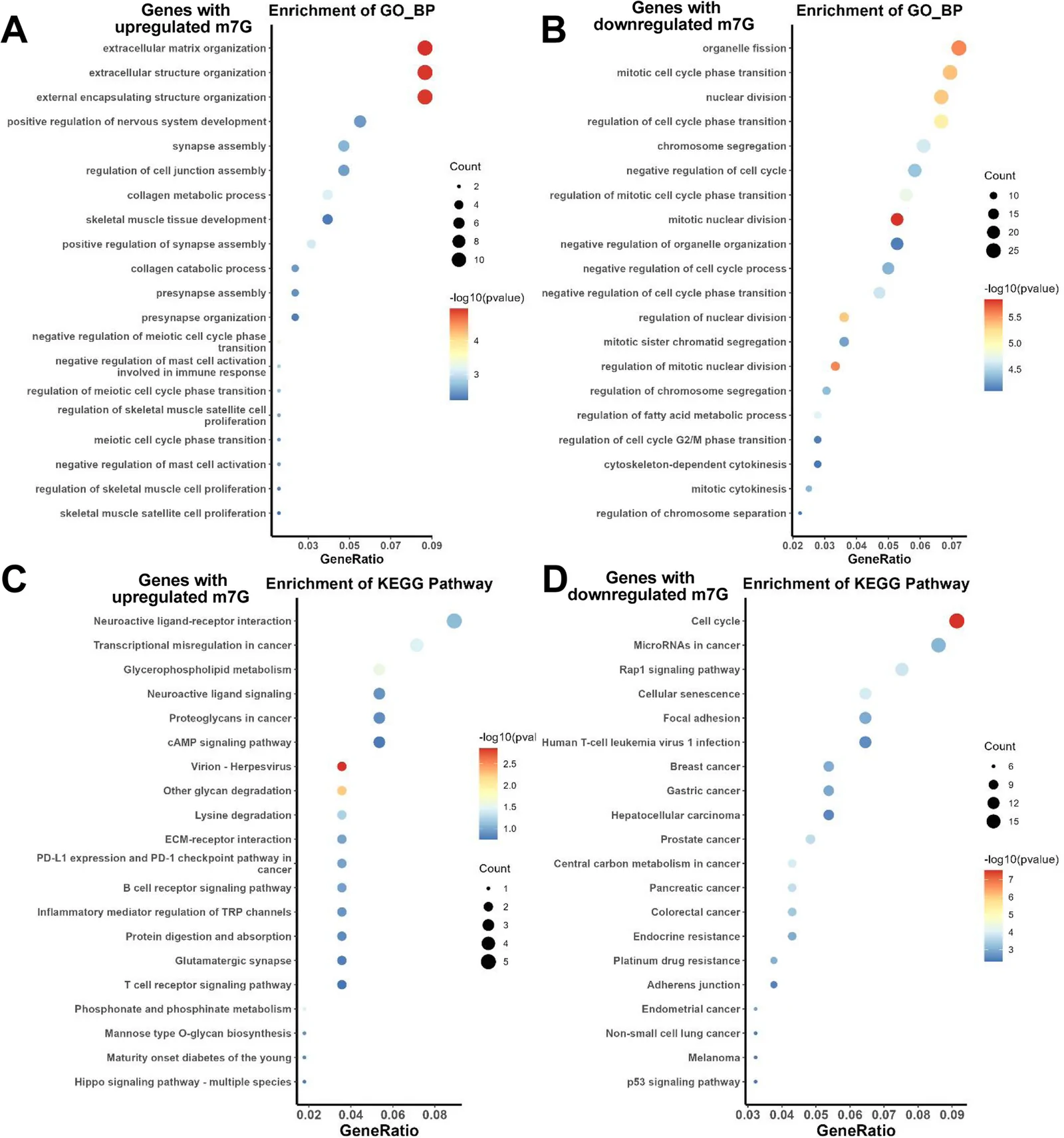
Functional analysis of genes with differential m7G peaks regulated by WDR4. **A**, **B** GO analysis of genes with differentially upregulated (**A**) and downregulated (**B**) m7G peaks regulated by WDR4. **C**, **D** KEGG analysis of genes with differentially upregulated (**C**) and downregulated (**D**) m7G peaks regulated by WDR4

To investigate the transcriptome-wide changes in m7G modification associated with WDR4 overexpression, we conducted m7G MeRIP-seq on macrophages with WDR4 overexpression and control. To characterize the m7G distribution pattern in WDR4-overexpressed and control macrophages, we examined the metagene profiles of all detected m7G peaks across the transcriptomes. This analysis demonstrated that m7G peaks were prominently located within coding sequences (CDSs) and 3’ untranslated regions (3’ UTRs) (Fig. 4A and B). Subsequently, motif analysis was conducted on all m7G peaks. We observed that the WDR4 overexpression group was found to be dominated by the GCGGCGGC motif, while the control group showed a prevalence of the CGGCGGCG motif (Fig. 4C). The Circos plot visually depicted the variations in m7G peaks throughout the genome (Fig. 4D). Figure 4E showed an enrichment of m7G modification around the transcription start site in the control and WDR4 overexpression groups. Subsequently, genes exhibiting the m7G modification were compared between the control and WDR4 overexpression groups. We observed that 2196 genes were exclusive to the control group, 23 were specific to the WDR4 overexpression group, and 455 were common to both (Fig. 4F). In comparison with the control group, 151 m7G peaks were upregulated and 403 peaks were downregulated in the WDR4 overexpression group (Fig. 4G). GO analysis revealed that genes with upregulated m7G modifications were chiefly involved in extracellular matrix organization, extracellular structure organization, and synaptic assembly, while genes with downregulated m7G modifications were mainly associated with negative regulation of the cell cycle and chromosome segregation (Fig. 5A and B). KEGG analysis indicated that genes with upregulated m7G modifications were mainly involved in the ECM-receptor interaction, cAMP signaling pathway, and T cell receptor signaling pathway, whereas genes with downregulated m7G modifications were primarily associated with the Rap1 signaling pathway and the p53 signaling pathway (Fig. 5C and D). These findings suggest that WDR4 extensively remodels the macrophage m7G methylome and regulates multiple biological pathways associated with inflammatory responses.

#### WDR4 enhances ZBP1 mRNA stability through m7G modification

**Fig. 6:**
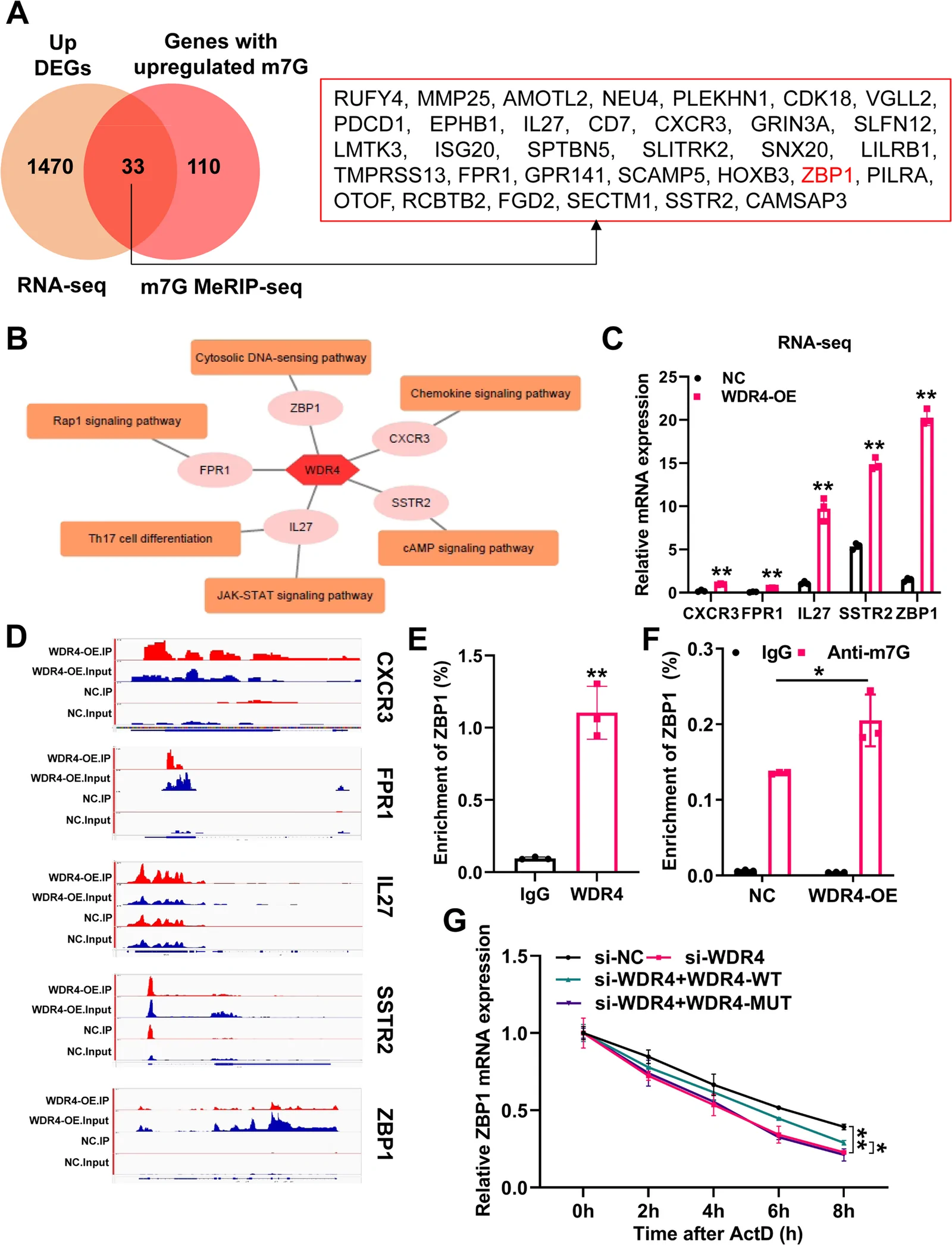
Screening of downstream genes of WDR4. **A** The Venn diagram illustrates the number of overlapping genes identified through m7G MeRIP-seq and RNA-seq analyses. **B** The WDR4-target genes-immune-related pathway network. **C** The expression of CXCR3, FPR1, IL27, SSTR2, and ZBP1 was assayed by RNA-seq. **D** The visualization of m7G modification peaks on CXCR3, FPR1, IL27, SSTR2, and ZBP1. **E** RIP-PCR assay was performed using an anti-WDR4 antibody to determine the binding enrichment of ZBP1 mRNA. **F** m7G-MeRIP-PCR assay was conducted to detect the m7G enrichment of ZBP1 mRNA in NC and WDR4-overexpressing macrophages. **G** ZBP1 mRNA stability was evaluated by actinomycin D chase assay followed by qRT-PCR analysis. Data are presented as mean ± SD (*n* = 3). *P < 0.05, **P < 0.01

To identify downstream targets of WDR4, we combined RNA-seq and m7G MeRIP-seq analyses to determine genes that had both upregulated differential expression and upregulated m7G modification. The Venn diagram showed that there were 33 intersection genes, including ZBP1 (Fig. 6A). The PPI network diagram showed the interactions among these 33 genes (Figure S1). To further identify the downstream genes of WDR4 that regulate macrophage polarization, we screened genes related to immunity. The pathways involved in CXCR3, FPR1, IL-27, SSTR2 and ZBP1 (Fig. 6B) are related to immunity [16–20], so these five genes were selected for subsequent research. The RNA-seq results showed that these five genes were significantly upregulated in the WDR4 overexpression group (Fig. 6C). The m7G modification peaks of these five genes were elevated in the WDR4 overexpression group compared to the control group (Fig. 6D). Previous studies have demonstrated that ZBP1 was involved in M1 macrophage polarization [21]. Therefore, ZBP1 was selected for further validation. RIP assays demonstrated a significant enrichment of ZBP1 mRNA in WDR4 immunoprecipitates compared with the IgG control (Fig. 6E). Furthermore, m7G MeRIP-qPCR analysis showed that WDR4 overexpression markedly increased the m7G modification level of ZBP1 mRNA relative to the control group (Fig. 6F). To determine whether WDR4-mediated m7G modification affects ZBP1 mRNA stability, RNA stability assays were performed following actinomycin D treatment. WDR4 knockdown significantly reduced ZBP1 mRNA stability, whereas re-expression of wild-type WDR4 restored ZBP1 mRNA stability. In contrast, WDR4 mutant failed to rescue ZBP1 mRNA stability (Fig. 6G). These findings indicate that WDR4 enhanced ZBP1 mRNA stability via m7G modification.

#### WDR4 suppresses the osteogenic differentiation of BMSCs by promoting ZBP1-associated M1 macrophage polarization

**Fig. 7:**
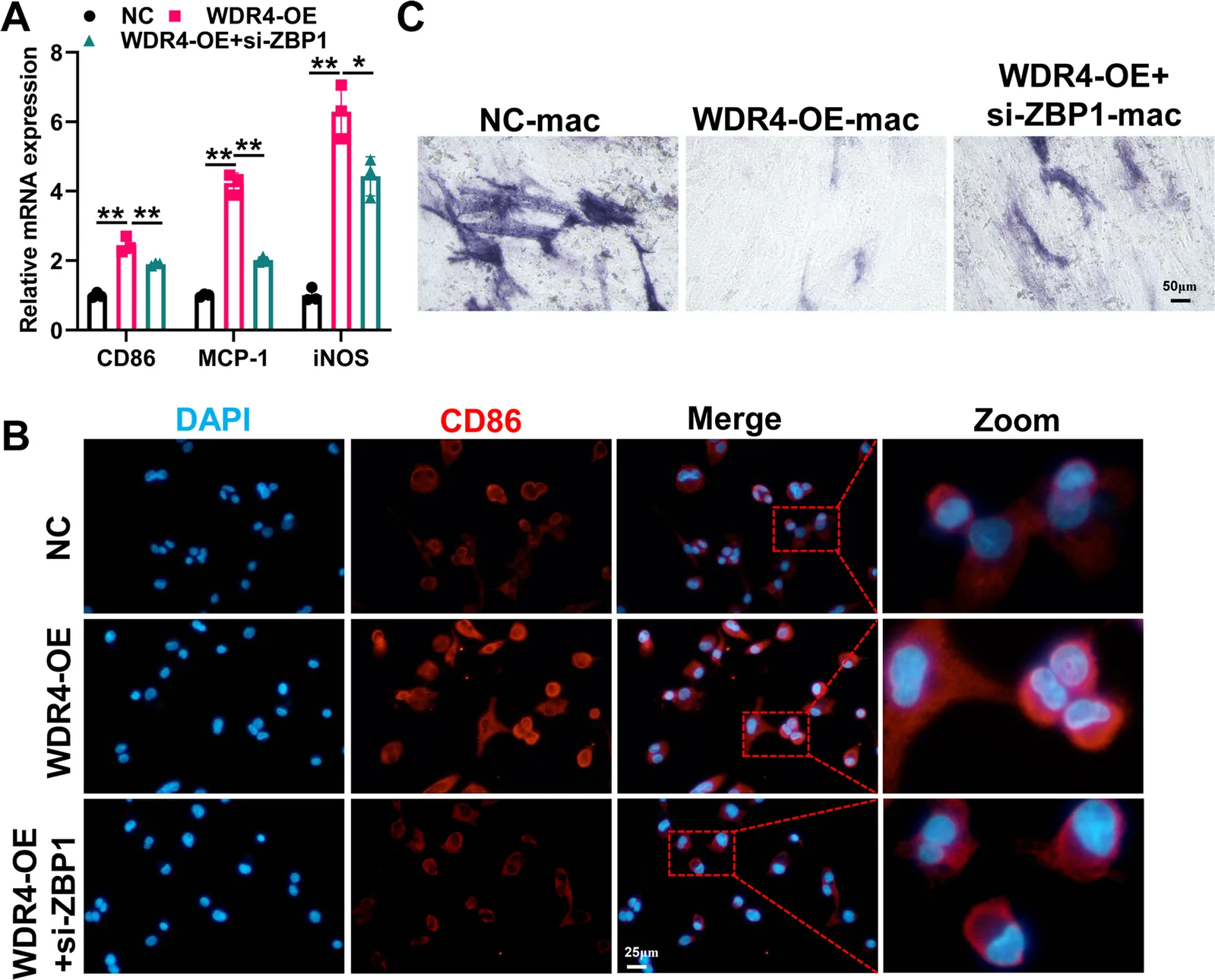
WDR4 inhibits BMSC osteogenic differentiation through ZBP1-associated macrophage M1 polarization. **A** qRT-PCR analysis of the M1 macrophage marker genes CD86, MCP-1, and iNOS in macrophages. **B** Representative immunofluorescence images of CD86 in macrophages. **C** Representative ALP staining of BMSCs co-cultured with macrophages in the indicated groups. Data are presented as mean ± SD (*n* = 3). *P < 0.05, **P < 0.01

To investigate whether ZBP1 mediates the regulatory effect of WDR4 on macrophage polarization, ZBP1 was knocked down in WDR4-overexpressing macrophages. qRT-PCR analysis showed that WDR4 overexpression markedly increased the expression of M1 macrophage markers CD86, MCP-1, and iNOS, whereas ZBP1 knockdown largely abolished this effect (Fig. 7A). Consistently, immunofluorescence staining demonstrated that CD86 expression was enhanced in WDR4-overexpressing macrophages, but was partially reversed following ZBP1 silencing (Fig. 7B). To determine whether WDR4-induced M1 macrophage polarization influences osteogenic differentiation, BMSCs were co-cultured with macrophages from the indicated groups. ALP staining revealed that co-culture with WDR4-overexpressing macrophages impaired ALP activity of BMSCs, whereas knockdown of ZBP1 largely restored ALP activity (Fig. 7C). These findings indicate that WDR4 promotes ZBP1-associated M1 macrophage polarization, thereby inhibiting the osteogenic differentiation of BMSCs.

## Discussion

**Fig. 8:**
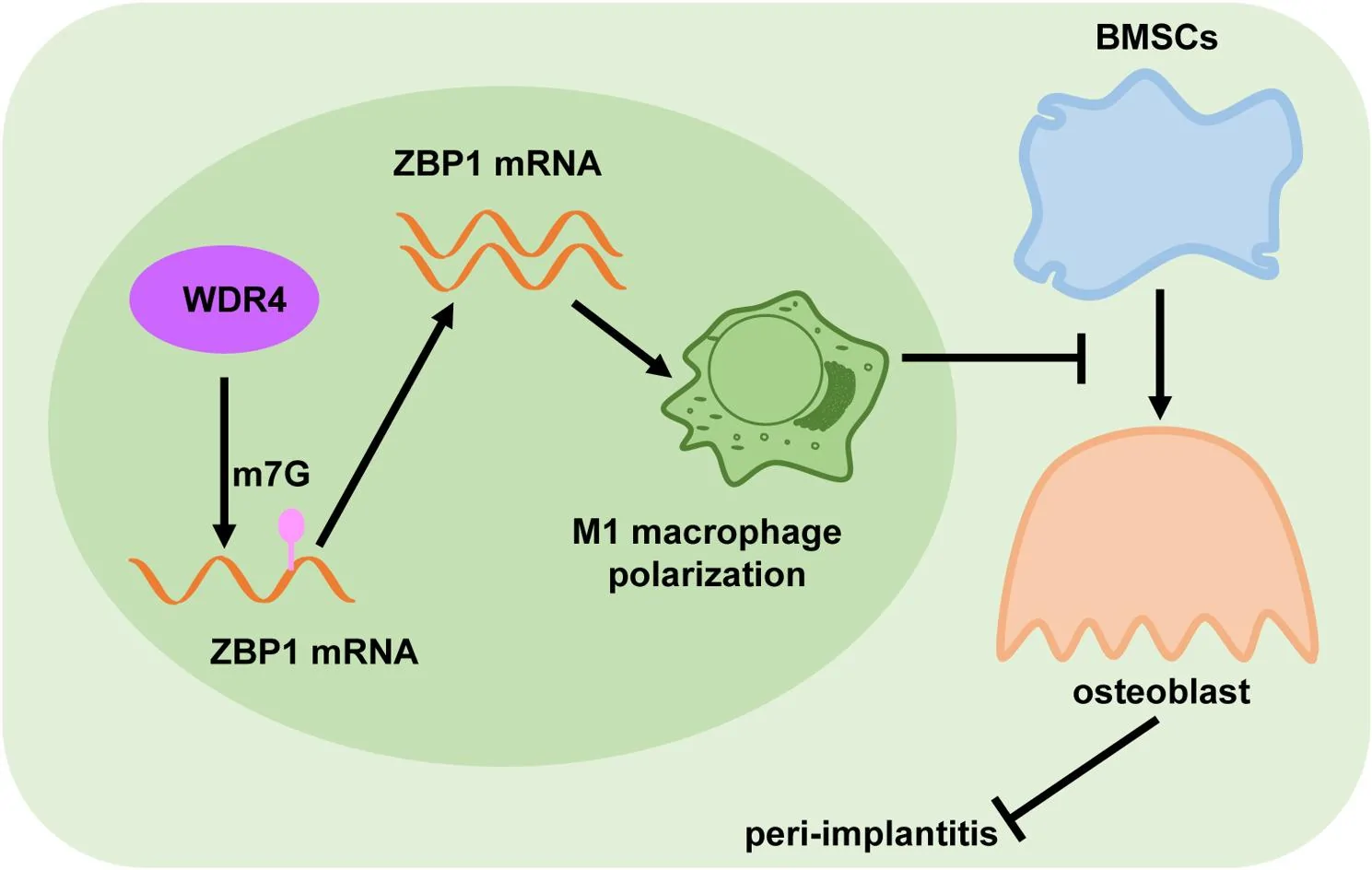
Working model by which WDR4 promotes peri-implantitis through inducing M1 macrophage polarization

RNA modifications play an important role in inflammatory diseases [22]. However, reports on the role of m7G regulators in peri-implantitis are scarce. In this study, WDR4 was identified as the only significantly upregulated m7G regulator in peri-implantitis tissues. Overexpression of WDR4 in macrophages promoted M1 polarization, thereby suppressing the osteogenic differentiation of BMSCs. Integrated analysis of RNA-seq and m7G-MeRIP-seq identified ZBP1 as a key downstream target of WDR4. WDR4 directly interacted with ZBP1 mRNA, enhanced its m7G modification, and increased its mRNA stability, resulting in elevated ZBP1 expression. Silencing ZBP1 markedly attenuated WDR4-induced M1 macrophage polarization and restored the osteogenic differentiation of BMSCs. Collectively, these findings demonstrate that WDR4 promotes peri-implantitis progression through the WDR4/m7G/ZBP1 axis (Fig. 8) and identify WDR4 as a promising therapeutic target for peri-implantitis.

RNA methylation is a common type of RNA modification, including m1A, m6A, m5C, m7G, and other modification forms, and exerts a vital role in oral diseases. The absence of METTL3 alleviated periodontal destruction and inflammation by increasing TNFAIP3 expression [23]. In a male rat model of apical periodontitis, FTO-related osteoclastogenesis promoted alveolar bone resorption through the HK1/USP14/RANK signaling pathway [24]. However, there are few reports on m7G modification in oral inflammatory diseases. In our study, we found that the m7G regulator WDR4 was significantly upregulated in the peri-implantitis group, and the peri-implantitis group showed a high level of m7G. Therefore, these findings suggest that WDR4 was involved in peri-implantitis through m7G modification.

Macrophage polarization plays a crucial role in peri-implantitis, and changes in its polarization state directly affect the development of inflammation [25]. Studies have shown that in the lesion tissues of peri-implantitis, M1 macrophages significantly increase [26]. These cells exacerbate the inflammatory response by releasing large amounts of pro-inflammatory factors and promote osteoclast activity, leading to the absorption and destruction of alveolar bone. In contrast, M2 macrophages are relatively scarce, making it difficult for inflammation to subside. Exogenous galactin-9 reduced the proportion of M1 macrophages and improved peri-implantitis [27]. The smallest-scale TiO2 honeycomb structure induced M2 macrophage polarization, thereby promoting implant-to-bone osteointegration [28]. Research indicated that m7G-related gene CYFIP1 was related with macrophage immune response [29]. Our research found that WDR4 promoted M1 polarization of macrophages, characterized by increased expression of CD86, MCP-1, and iNOS. Therefore, WDR4 is a key target for the treatment of peri-implantitis.

The meticulous coordination between macrophages and BMSCs is crucial for bone remodeling [30]. In the inflammatory microenvironment around implants, immoderate infiltration of M1 macrophages and inflammatory factors confines the osteogenic differentiation of BMSCs, leading to tissue damage [31]. Hence, strategies that regulate hyperactive M1 macrophage polarization and promote M1-to-M2 repolarization to initiate the next phase of bone remodeling have garnered significant attention in the field of inflammatory bone defects. Overexpression of BMAL1 in bone marrow-derived macrophages could inhibit the TLR2/NF-κB signaling pathway, reduce pyroptosis, lower the M1/M2 ratio, and thereby promote the osteogenic differentiation of BMSCs [32]. In periodontitis, macrophages mediated inflammatory stimulation via the exosome pathway, thus suppressing the osteogenic differentiation of BMSCs [33]. In this study, co-culture with WDR4-overexpressing macrophages significantly downregulated osteogenic differentiation markers OPN, RUNX2, BMP2, and ALP, and reduced ALP activity in BMSCs. In summary, WDR4 regulates the osteogenic differentiation of BMSCs through macrophage polarization.

m7G modification affects gene expression in key inflammatory signaling pathways by regulating the stability, localization and translation efficiency of RNA. TMEM11 interacted with METTL1, enhancing the m7G methylation of Atf5 mRNA, thereby increasing Atf5 expression, which ultimately promoted cardiac repair [34]. METTL1 promoted post-ischemic angiogenesis via VEGFA mRNA translation dependent on m7G methylation, highlighting a novel avenue for addressing ischemic diseases [35]. METTL1 increased the stability and translation of Sptbn2 through m7G methylation, thereby inducing neurogenesis and exerting an anti-Alzheimer’s disease effect [36]. In this study, we identified ZBP1 as a key downstream target of WDR4 and demonstrated that WDR4 enhanced the m7G modification and stability of ZBP1 mRNA. Previous studies have shown that ZBP1 is associated with macrophage polarization, and the M1 marker decreases after ZBP1 knockout [21]. In agreement with it, our experiments demonstrated that knockdown of ZBP1 reversed WDR4-induced M1 macrophage polarization and rescued the impaired osteogenic differentiation of BMSCs.

This study has several limitations. First, our in vitro findings were obtained using THP-1-derived macrophages and require further validation in primary macrophages. Second, a sham ligature control was not included in the animal model, preventing a clear distinction between the effects of mechanical trauma and Porphyromonas gingivalis infection. Future studies addressing these limitations will further strengthen our findings.

In conclusion, WDR4 promotes the M1 polarization of macrophages and inhibits the osteogenic differentiation of BMSCs through m7G modification, ultimately leading to peri-implantitis. Our findings imply that WDR4 may be a promising target for therapeutic treatment of peri-implantitis.

## Supplementary Information

Supplementary Material 1.

Supplementary Material 2.

Supplementary Material 3.

## Data Availability

The data sets used and/or analyzed during the current study are available from the corresponding author on reasonable request.
