# Supplementary material for: WDR4-mediated m7G modification of ZBP1 mRNA drives peri-implantitis by promoting M1 macrophage polarization
Source: Hereditas. 2026 Aug 14;163:129. doi: 10.1186/s41065-026-00723-5 (PMC13756913; doi:10.1186/s41065-026-00723-5)
Supplement: Supplementary file 1 [file 41065_2026_723_MOESM1_ESM.docx]

**Table S1 Primer sequences.**

| **Primers** | **Sequence (5' to 3')** |
| --- | --- |
| WDR4-F | TAACCGATGACAGTAAGCGTCT |
| WDR4-R | TCTCCTCCGAGGCTATGAAAG |
| CD86-F | CTGCTCATCTATACACGGTTACC |
| CD86-R | GGAAACGTCGTACAGTTCTGTG |
| MCP-1-F | CAGCCAGATGCAATCAATGCC |
| MCP-1-R | TGGAATCCTGAACCCACTTCT |
| iNOS-F | AGGGACAAGCCTACCCCTC |
| iNOS-R | CTCATCTCCCGTCAGTTGGT |
| OPN-F | CTCCATTGACTCGAACGACTC |
| OPN-R | CAGGTCTGCGAAACTTCTTAGAT |
| RUNX2-F | GAATGCTTCATTCGCCTCACA |
| RUNX2-R | GTGACCTGCGGAGATTAACCA |
| BMP2-F | ACTCGAAATTCCCCGTGACC |
| BMP2-R | CCACTTCCACCACGAATCCA |
| ALP-F | ACAAGCACTCCCACTTCATCTG |
| ALP-R  ZBP1-F  ZBP1-R | GGTCCGTCACGTTGTTCCTG  GGGAATGAGGACAGCAAAAG  CAGAATCTTCTGGGCGGTAA |
| GAPDH-F | ACAACTTTGGTATCGTGGAAGG |
| GAPDH-R | GCCATCACGCCACAGTTTC |
