# Supplementary material for: WDR4-mediated m7G modification of ZBP1 mRNA drives peri-implantitis by promoting M1 macrophage polarization
Source: Hereditas. 2026 Aug 14;163:129. doi: 10.1186/s41065-026-00723-5 (PMC13756913; doi:10.1186/s41065-026-00723-5)
Supplement: Supplementary file 2 [file 41065_2026_723_MOESM3_ESM.docx]

Figure 1H WDR4

43kDa

WDR4

Figure 1H GAPDH

36kDa

GAPDH

Figure 2C WDR4

WDR4

43kDa

Figure 2C GAPDH

36kDa

GAPDH
